# Historical influence on the practice of chiropractic radiology: Part I - a survey of Diplomates of the American Chiropractic College of Radiology

**DOI:** 10.1186/s12998-017-0146-y

**Published:** 2017-05-08

**Authors:** Kenneth J. Young

**Affiliations:** School of Arts, School of Health Professions, Murdoch University, Perth, WA 6150 Australia

## Abstract

**Background:**

It is known that not all chiropractors follow mainstream guidelines on the use of diagnostic ionising radiation. Various reasons have been discussed in the literature, including using radiography to screen for congenital anomalies, to perform postural analysis, to search for contraindications to spinal manipulation, and to document chiropractic subluxations, i.e., tiny anatomical displacements of vertebrae thought to affect nerves and health. The visualisation of subluxations was the reason chiropractic first adopted the x-ray in 1910. There has never been a study of the influence of this historical paradigm of radiography on the practices of chiropractic radiologists (DACBRs or Diplomates of the American Chiropractic College of Radiology).

**Methods:**

A survey was administered with a modified Dillman method using SurveyMonkey and supplemented by hard copies distributed at a professional conference. The target population was all active DACBRs. There were 34 items, which consisted of multiple choice and open-ended interrogatives on all three areas in which chiropractic radiologists work: education, clinical practice, and radiology practice.

**Results:**

The response rate was 38% (73 of 190 DACBRs). Respondents reported that the historical paradigm of radiography was found in all areas of practice, but not as a major aspect. The majority of respondents did not condone that historical paradigm, but many tolerated it, particularly from referring chiropractors. Radiographic subluxation analysis was reportedly perpetuated by private clinical practitioners as well as technique instructors and supervising clinicians in the teaching institutions.

**Conclusions:**

Within the chiropractic profession, there is a continuing belief in radiographically visible subluxations as a cause of suboptimal health. This situation is sustained in part due to the reticence of other chiropractors to report these practices to licensing and registration boards. Investigation into other structures supporting a vitalistic belief system over science in chiropractic is recommended. In addition, it may be useful to explore remunerative systems that move beyond the inherently conflicted fee-for-service model.

**Electronic supplementary material:**

The online version of this article (doi:10.1186/s12998-017-0146-y) contains supplementary material, which is available to authorized users.

## Background

BJ Palmer first introduced radiography to the profession of chiropractic in 1910. His idea was that the new technology would allow visualisation of tiny misalignments of vertebrae that were thought to impinge on nerves and affect transmission of ‘mental impulses’. He called this abnormal state ‘subluxation’ and cited it as the ultimate cause of ill health [1]. This belief is perpetuated in segments of the profession today; there are chiropractors that vigorously argue for the practice of routine radiography [2]. Belief in the visibility of chiropractic subluxations on plain radiographs has helped lead to overuse of x-ray imaging. Among other factors, such as screening for congenital anomalies, performing postural analysis, and searching for contraindications to spinal manipulation, some chiropractors choose not to follow mainstream guidelines on the use of ionising diagnostic radiation because of this historical belief system. Chiropractic techniques systems associated with this practice use subluxation identification and quantification, post-course-of-care radiography, postural analysis, and/or full spine imaging [3]. In a survey of chiropractors in the United States of America (USA) Harger, et al. [4]. found that 37% of their respondents thought the most important use of radiography was to establish the presence of subluxation. They also found that 43% of respondents radiographed more than 25% of their patients, a figure well above that of mainstream usage. Four percent responded that establishing a clinical/pathological diagnosis never justified the use of radiographs. Two Canadian studies found that up to 59% of chiropractors would radiograph patients with acute low back pain even if they had no ‘red flag’ clinical indications to justify the use of ionising radiation [5, 6]. Ammendolia et al. [7] reported a disparity between mainstream radiography guidelines and the usage of radiography in the outpatient chiropractic clinics at some teaching institutions. They found various reasons not supported by guidelines cited to justify using x-rays, including screening for contraindications to spinal manipulation and providing assurance to patients who expressed anxiety about their back pain. They also reported that several schools used no guidelines at all.

Diplomates of the American Chiropractic College of Radiology (known as DACBRs or chiropractic radiologists) are a group of chiropractors who have undergone additional training in diagnostic imaging after attaining a chiropractic qualification. Currently this means attending a three-year full-time residency at a chiropractic teaching institution then passing written and oral board examinations. Upon successful completion, they become members of the American Chiropractic College of Radiology (ACCR). The ACBR is the only organisation in the world that certifies chiropractors in diagnostic imaging. The analogues of these groups in the medical community are the American Board of Radiology (ABR) and American College of Radiology (ACR). This chiropractic ‘speciality’ is not officially recognised by licensing or registration boards but DACBRs have been shown to be competent at musculoskeletal imaging, [8] and are esteemed within the chiropractic community, with some chiropractors electing to have all their radiographs interpreted by a DACBR [4]. Chiropractic radiologists work across the spectrum in chiropractic, reporting on images, teaching, and working in clinical practice treating patients [9–11]. Because of their special interest in radiology and their penetration into all areas of chiropractic, DACBRs have understanding and insight into the paradigms chiropractors used for radiography in the field, how those paradigms relate to evidence-based practice, and what effect they might be exerting on the practices of chiropractors who focus on diagnostic imaging. A review of the literature found no studies that focused on the historical paradigm of radiography and its lingering influence on DACBRs. The purpose of this study was to explore the influence of the historical chiropractic radiography paradigm on the current practice of chiropractic radiologists.

## Methods

Approval was obtained from the Murdoch University Human Research Ethics Committee (approval number 2015/142). The study group consisted of all current members of the ACCR (*n* = 190). The survey instrument consisted of 34 multiple-choice and/or open-ended items with free-text answer boxes. The items were designed to elicit responses for three areas of practice, radiology reporting, academics, and clinical practice. Basic demographic data were also collected, including age, sex, and continent of residence. A draft copy of the questionnaire was sent to three chiropractic radiologists to edit for clarity and face validity. Radiology practice was defined as reporting on diagnostic images for chiropractors. Clinical practice was defined as treating patients directly. Teaching meant full- or part-time instruction in any capacity at a chiropractic teaching institution. Items about technique systems, full-spine radiography, and post-adjustment/post course-of-care radiography were included as these procedures are associated with chiropractic technique systems that use radiography for subluxation analysis [12]. The main idea of the survey was to get at the reasoning behind some of the issues that DACBRs deal with in their various roles. Since not all DACBRs work in all roles or perform all possible radiology-related functions, there were many items with ‘skip ahead’ instructions, depending on answers to a current item. For instance, after the demographics section, the survey enquired about teaching. If a respondent did no teaching as a DACBR, the instruction was to skip ahead to the next section, clinical practice. In addition, if a respondent answered ‘no’ to a particular professional activity, and the follow-up question was about the rationale behind performing that activity, the respondent was instructed to skip the follow-up question. Please see Additional file 1 for the survey instrument in its entirety.

An internet-based, anonymous survey using SurveyMonkey was implemented. The online distribution methodology was based on a modified Dillman method [13]. First, a herald notice was sent to inform the recipients of the purpose of the study, stimulate their interest and ask for their cooperation. One week later the questionnaire was opened on the internet. Then an online survey link was sent directing the recipient to the Information Letter, Informed Consent, and the questionnaire. Two fortnightly reminders followed. Those recipients who have already completed the questionnaire were asked to ignore the reminders. The Secretary of the ACCR sent all the emails to the ACCR members. By coincidence, the annual conference of the ACCR occurred during the time allotted to data collection, and the author was able to attend. Hard copies of the survey were printed and one announcement was made to the conference attendees requesting that they fill in a hard copy if they had not already done an online version. A stack of questionnaires were left at the registration desk of the conference, and the receptionists agreed to distribute blanks and collect completed forms in order to maintain anonymity of respondents.

Response numbers and percentages were calculated. Themes in open-ended responses were identified and commonalities with the historical paradigm of radiography were noted.

## Results

Internet questionnaires were completed by 64 respondents; nine hard copies were completed and returned, for a total of 73 and a response rate of 38%. Many of the questionnaires were partially incomplete but all questionnaires were included in the analysis. The last item was an open-ended item that elicited some lengthy responses, and therefore is considered in a separate paper (Part II).

Table 1 displays the demographic details of respondents. Most were male (75%), middle-aged (36% were 45–54 years old) and lived in North America (92%). The respondents included 39 people (53%) employed at a chiropractic teaching institution. 13 (20%) were in clinical practice. Fifty-eight DACBRs (81%) were in radiology practice. The total was greater than 100% because many worked in two or more areas.

**Table 1:** Demographic characteristics of respondents (Items 1, 2, 3)

Sex (*n* = 72)	Female	18
	Male	54
	Other	0
Age (*n* = 72)	Under 25	0
	25–34	5
	35–44	15
	45–54	26
	55–64	19
	65 and over	8
Location (*n* = 72)	Australasia	3
	Europe	2
	North America	67
	United Kingdom	0

### Teaching

Item 4 (*N* = 73) asked: ‘Are you currently employed in any capacity by a chiropractic teaching institution?’ 39 reported affirmatively, 34 negatively. Item 5 (*N* = 39) found that sixteen respondents (41%) reported that radiographic subluxation analysis was taught at their institutions. Item 6 (*N* = 16): ‘By whom is (are) the system(s) taught at your institution?’ No DACBRs reported teaching any of these systems, and indicated that others at the institutions taught them: technique instructors (*N* = 14), adjunct/casual faculty/staff (*N* = 2), clinic supervisors (*N* = 2). Item 7 asked which systems were taught and requested a rationale for the system(s), if known. Usually more than one system was taught at any one institution. See Table 2 for details.

**Table 2:** Technique systems taught and rationale for them (Item 7, *N* = 12)

Response number	Technique name(s)	Rationale
1	Gonstead	None given.
2	Basic, Gonstead, Upper Cervical	These courses have historically been included in the curriculum.
3	Gonstead, Unknown Toggle	Unknown.
4	General lines of mensuration are taught such as rotation of the ilium (internally rotated, externally rotated), rotation of vertebra, etc. No specific system is used as far as I’m aware.	N/A
5	Upper cervical specific, Gonstead line analysis	History. This is what has been taught for the last half century.
6	We have approximately 6 upper cervical techniques, Gonstead and CBP (Chiropractic BioPhysics) that require x-ray marking as part of the technic.	None given.
7	Blair, Gonstead, upper cervical	They are included because someone in administrative power feels they are relevant and worth teaching to the future profession. My guess anyway.
8	Palmer Upper Cervical Technique, Grostic, elective course Pettibon, elective course Gonstead	None given.
9	Gonstead, Diversified, Basic technique	None given.
10	Gonstead (required course), upper cervical (elective)	Demand and tradition.
11	Gonstead, CBP. Not taught in the core curriculum, offered as elective/selective courses.	None given.
12	NUCCA (National Upper Cervical Chiropractic Association), Atlas Orthogonal (Epic), Upper cervical knee chest, Gonstead, Toggle, Blair, CBP	[This institution] has a huge upper cervical culture and correction of misalignments is very important to those techniques.

The most commonly mentioned system was Gonstead at *N* = 9, followed by (Logan) Basic (*N* = 3), Upper Cervical (unspecified, *N* = 3), CBP (*N* = 2), and Blair (*N* = 2), with all other systems only being mentioned once.

Item 8 (*N* = 38): ‘Have you ever refused a request to teach a chiropractic radiographic subluxation analysis system in a chiropractic teaching institution?’ Five responded ‘yes’ and 33 ‘no’. Item 9 (*N* = 7) asked for an explanation and outcome of the refusal. Two of the seven volunteered that they had never been asked, hence the disparity between five positive responses to Item 8 but seven responses to its follow-up, Item 9. One stated ‘this is not my role.’ One had been asked to teach full-spine and responded, ‘I refuse to teach or encourage the use of full-spine radiographic analysis.’ One stated, ‘To my knowledge, there is not sufficient evidence to support the practice of detecting/diagnosing subluxation on radiographs.’ One stated, ‘I have tried for decades to teach a functional approach to spinal evaluation.’ One indicated leaving a job on these grounds:

I refused to teach it in the clinic system at a prior institution on the grounds that I do not know how to do it, and that it would be an insult to those who are certified in the technique for me to teach the system (Gonstead). I prevailed. I probably would not have prevailed had I simply stated “I refuse to teach a system of radiographic interpretation that is founded in fantasy-land. I won’t risk my personal reputation by instructing our future chiropractors in antiquated stupidity.” However, as the institution was adopting this sort of approach to our profession, I moved on to a science based institution.

Item 10 (*N* = 38) enquired as to whether radiographic subluxation analysis was performed on patient images in a teaching institution’s clinic. Eight said ‘yes’; 30 ‘no’. The following question (Item 11) asked who taught the systems. More than one answer was possible per respondent, and a text box was provided for answers not in the list. The list consisted of ‘self’, ‘other chiropractic radiologists’, ‘technique instructors’, ‘clinic supervisors’, and ‘adjunct/casual faculty/staff’. See Table 3.

**Table 3:** By whom is the use of chiropractic radiographic sybluxation analysis systems used on patient in your institution’s clinic? (Item 11, *N* = 8)

Response number	Role of person(s) teaching a system	Number of times the role was given as a response
1	Clinic supervisors	6
2	Technique instructors	2
3	Adjunct/casual faculty/staff	1
4	Case approval doctors	1
5	Student interns	1
6	Students occasionally – one or two clinic supervisors using specialty techniques	1
7	Anyone who evaluates the film, this is a chiropractic college.	1
8	Interns	1

Item 12 (*N* = 8) asked for the names of systems used in the institution’s clinic and a rationale for their use. The same systems as in Item 7 were cited with two exceptions. One respondent stated, ‘I have my own analysis procedures and frequently interact with technique instructors, clinic faculty, and interns.’ Another noted a brand name of equipment that had been donated to the institution. This donation served as the rationale for its use in the clinic. The only other rationales given were ‘clinician preference’ (*N* = 1) and ‘Students learn systems in the classroom. They are not actively discouraged from using them in the clinic, however it is not a routine part of clinical care’ (*N* = 1).

Thirty people answered Item 13 about post-adjustment or post course-of-care radiographs being taught as part of the curriculum. Three respondents indicated that it was. Twenty-six of them indicated in the negative, and one was unsure. Item 14 was the follow up question enquiring who taught the system(s), and the item allowed more than one answer to be given; in addition a text box was provided for further answers. Three people responded, and technique instructors were indicated twice, clinic supervisors once, and adjunct/casual faculty/staff once. One person responded ‘no one’. Item 15 (*N* = 4) asked for the rationale behind this use of radiography. The responses were as follows: ‘not included in the curriculum’, ‘technique specific’, ‘unknown’, and one indicated that it was because the technique system was not part of the core curriculum, but rather an elective.

Item 16 (*N* = 30) asked about post-adjustment or post course-of-care radiographs being used in their institution’s clinic. Three respondents indicated positively, 26 negatively. Item 17 (*N* = 2) asked who performed the procedure on patients. More than one answer was possible. Both indicated that clinic supervisors performed the process. The rationale for the procedure was enquired about in item 18. It was unknown for one respondent; one wrote ‘technique specific’, and one wrote ‘not included in the curriculum’.

### Clinical practice

Twelve respondents stated that they worked in clinical practice, 52 that they did not (item 19, *N* = 64). Two indicated that they used a system of radiographic subluxation analysis (Item 20, *N* = 24). On the follow-up question, Item 21 (*N* = 5), one respondent indicated that the technique systems used were Chiropractic Biophysics and Diversified. One wrote ‘standard radiographic mensurations’; one wrote ‘standard ACR [American College of Radiology] terminology. One wrote ‘N/A’. One provided a definition of subluxation:

Subluxation: partial dislocation of a joint or articulation in which some portion of the articular surfaces remain intact. Radiographically may demonstrate dysfunction and/or instability on functional studies and clinically. MRI may demonstrate internal derangement of the joint that may include some or all of the following; articular cartilage disruption, articular capsule deformity or disruption, synovial tags or inflammation, synovial cysts, intra-articular ligamentous deformity or disruption, and excessive fluid within the joint capsule and joint capsule.

### Radiology practice

Seventy-two respondents answered Item 22 as to whether they worked in radiology practice. Fifty-eight responded positively, 14 negatively. Item 23 (*N* = 58) enquired about reporting on full-spine images and 48 indicated that they did. The results for Item 24 are in Table 4. Note that although 48 people responded that they reported on full-spine images, 49 responded to the following question, ‘What percentage of your practice is comprised of reporting full-spine images?’

**Table 4:** Percentage of reporting on full-spine radiographs (Item 24, *N* = 49)

Percentage of practice that reporting on full-spine images comprises	Number of positive responses	Percentage of respondents responding positively to this item
<20%	35	71%
20–39%	7	14%
40–59%	2	4%
60–79%	2	4%
80–100%	3	6%

Fifty-five DACBRs answered Item 25: ‘Regarding justification for the use of ionizing radiation, what percentage of the patients referred to you reflects the use of mainstream radiographic guidelines?’ Most respondents indicated that most of their referral information reflected the use of mainstream radiography guidelines. Only two responded that less than 20% of their referrals reflected the use of guidelines. Table 5 shows the results to that item.

**Table 5:** Percentage of referrals to DACBRs that reflect use of mainstream radiographic guidelines (Item 25, *N* = 55)

Percentage of practice that reflects use of guidelines by referrers	Number of responses	Percentage of respondents
<20%	2	4%
20–39%	5	7%
40–59%	10	4%
60–79%	12	22%
80–100%	26	47%

Forty-two respondents answered Item 26: ‘What are some of the justifications you see on referral forms that do NOT reflect the use of mainstream radiographic guidelines?’ As this was an open-ended question, more than one answer was possible per respondent. Table 6 shows the results to that item.

**Table 6:** Justifications for diagnostic imaging that do not reflect the use of mainstream guidelines received by DACBRs from referring chiropractors (Item 26, *N* = 42)

Response	Number of respondents
No red flags	4
No history	5
Equivocal exam findings	1
Uncomplicated back or neck pain	6
Subluxation analysis	4
No symptoms	2
Allergies	1
Postural analysis (not for scoliosis)	3
‘Rule out pathology’	3
Looking for anomalies	1
‘Has never been x-rayed before’	1
‘Scoliosis assessment’ in patient with straight back	1
Failure to use Ottawa ankle/knee rules	2
Routine for motor vehicle accidents	1
‘Something just isn’t right’	1
Rule out contraindications to adjustment	3
‘Tightness’ or ‘soreness’	1
Minimal trauma	2
Full spine films on every patient without regard for symptoms or age	1
Areas imaged do not correlate to symptoms	5
Young person being assessed for degenerative changes	1
Repeat imaging due to recent prior imaging not being available	1
No clear or specific justification	2
Rule out disc herniation	1
Segmental dysfunction	1
‘Chiropractic evaluation’	1
‘Positive posterior lumbar instability test, rule out spondylolisthesis’	1
Postural change over time	1
Post treatment	2
‘Wellness care’	1
‘3 region subluxations’	1
Lumbar oblique images for intervertebral foraminal stenosis	1
Follow up on scoliosis well past skeletal maturity	1

Fifty-five respondents answered Item 27, which asked if they reported on images from referrers that they knew took radiographs on all or nearly all their patients. Thirty-nine indicated that they were aware they had done so. Sixteen answered that they were not. The follow-up (Item 28, *N* = 45) provided a list of possible answers. More than one answer per respondent was possible. This item addressed the way they dealt with the situation: ‘Reporting on the images of chiropractors who radiograph all their patients may present an ethical dilemma to the reporting chiropractic radiologist. How do you deal with this issue?’ Forty-five respondents answered. See Table 7.

**Table 7:** Rationales for dealing with referring chiropractors who are known to image all or nearly all their patients (Item 28, *N* = 45)

Response	Number of responses
I don’t have a problem with this practice	9
I know that the images are at least being properly scrutinized for pathology	30
It’s not my place to question another chiropractor’s clinical judgment	19
I do not speak with or examine the patients, so I’m not in a position to pass judgment	27
It’s just part of the business I’m in	11
I have raised the issue with the referring chiropractors, but none have changed	9
I have raised the issue with the referring chiropractors, and have helped reduce this practice	12
I have reported such practices to licensing/registration/public health boards	2

This item also included a text box referrers could use to list other rationales or to elaborate further. The majority of answers given were elaborations rather than different rationales. However, one DACBR indicated that complaining would only serve to reduce referrals. The full text responses for Item 28 are found in Additional file 2.

Item 29 (*N* = 57) asked if DACBRs used any chiropractic radiographic subluxation analysis systems in their radiology practice. Five answered affirmatively, 52 negatively. The follow-up question (Item 30, *N* = 7) asked which systems they used and included a text box for the rationale. As in Items 8 and 9, the two negative responses to the follow-up, ‘not applicable’, and ‘not really’, account for the greater number of responses to the follow-up than the original question. The full texts of the responses to Item 30 are in Table 8.

**Table 8:** ‘Which system(s) do you use and why?’ (Item 30, *N* = 7)

Respondent	Response
1	Chiropractic BioPhysics. I only use the cervical and lumbar lordosis angles for film reading clients.
2	Not applicable
3	Show me a radiologist with a ruler and I will show you a radiologist in trouble. Systems are for teaching/learning process and useful for that purpose.
4	Not really.
5	Define ‘subluxation.’ Lines of skeletal measurements are used.
6	Marking systems available on [brand name] and e-film systems.
7	Diversified, CBP. Medicare and work comp want the word ‘subluxation’ to justify care and help chiropractors provide chiropractic.

Item 31 (*N* = 56) asked if DACBRs had ever been asked to use a chiropractic radiographic subluxation analysis system by a referrer, and what their response was to the request. Fifty-six answered this item; eighteen indicated positively, 38 negatively. The responses to the open-ended portion of the item are in Table 9.

**Table 9:** Explanation of response to a request to use a radiographic subluxation analysis system (Item 31, *N* = 18 for this portion of the question)

Response	Number of times this response appeared
Flat refusal	7
Refusal by declining knowledge of the techniques being requested	5
Acceded to the use of Medicare/Workers Compensation definitions	2
Conditionally acceded, ‘depending on the time required to do the reports’	1
Unconditionally acceded	1
Acceded to measuring cervical lordosis	1
‘Never been asked’	1

One respondent who was classified above as a ‘flat refusal’ elaborated: ‘I can report on biomechanics as depicted. I believe malfunction is what defines what we call subluxation – as compared to the classic determination of the word.’ Another stated, ‘I only use what I can find in Keats’. Another stated, ‘I apologize, however I do not utilize radiographic subluxation analysis systems. I read the case for pathology and then you can draw whatever lines you want.’ One respondent wrote that the referring chiropractor who was refused continued to refer images for reporting. Two others referred the subluxation analysis back to the referrer, indicating that the referrer had been trained in the particular system and so should be the one to use it. One respondent who acceded wrote, ‘Only on that ridiculous Federal Workers’ Comp system that requires it.’

Fifty-eight respondents answered Item 32 enquiring as to whether they report on post-treatment radiographs as far as they are aware. Seventeen answered positively; 41 negatively. Item 33 (*N* = 34) enquired as to the percentage of their reports was for post-treatment or post-course of chiropractic care radiographs. Thirty-three responded that those reports comprised less than 20% of their practice, one indicated it represented between 20 and 39% of his/her practice. One practitioner clarified that it was only under circumstances of a patient failing to respond to treatment as expected that they reported on post-treatment radiographs. This clarification was written on a hard-copy version of the survey even though no text box was provided. T﻿he resul﻿ts of Item 34 are reported in Part II of this study.

## Discussion

This survey explored the effect of the historical paradigm of radiography in chiropractic on the current practices of DACBRs. The traditional concept of chiropractic subluxation as a lesion at the root of ill health is not supported by evidence, and neither is the idea that a radiographically demonstrable displacement of vertebrae is an essential component of this ‘lesion’ [14–26]. The hypothesis of this paper was that the historical chiropractic paradigm of radiography, to visualise subluxations, was still exerting an influence on the practices of chiropractic radiologists. The hypothesis was supported by the findings, although none of the respondents reported that it was a dominant element in their practices.

All health professions, including chiropractic, are experiencing increasing pressure around the world to align with evidence-based standards of care, and this includes adhering to accepted standards for radiography. In the UK, one of the most common causes of a complaint investigated by the General Chiropractic Council is unjustified or excess radiography [27]. Johnson noted that if the chiropractic profession does not enforce guidelines and protocols itself, an external entity will do so [28]. One component of a profession, as opposed to a trade, is self-regulation; it is the freedom exchanged for fulfilling the fiduciary contract that places patients’ interests above those of the practitioner [29]. Therefore, complying with mainstream, evidence-based guidelines for radiography is imperative to the profession.

Epistemologies for radiography such as appeal to tradition or appeal to authority are unacceptable. Three respondents to the item about their teaching institution’s rationale for teaching radiographic subluxation analysis answered ‘history’, ‘tradition’, or that it was an administrator’s preference. Similarly, justifications such as ‘wellness care’, ‘subluxation analysis’ and ‘chiropractic evaluation’ reported in this study are also not valid, but do invoke the historical paradigm of radiography. One respondent indicated that a system of radiographic subluxation analysis was being used at a teaching institution simply because equipment enabling it had been donated. This would seem to fit the epistemology of either appeal to novelty or appeal to popularity, rather than evidence-based healthcare.

Marchiori, Hawk and Howe indicated that guidelines for radiography used by medical doctors may not readily transfer to the chiropractic profession, due to differences in methods of treatment, but did not elaborate further [9]. A similar idea was cited by respondents to the current survey, (e.g., ‘I also think chiropractors who put force into the spine are justified to image what they push on…’) It is possible that there may be some biomechanical justifications for radiography unique to chiropractors, but these must be researched and documented to a reasonable level of certainty before abandoning current guidelines on the use of ionising radiation. This has not been done within the profession. The few published papers advocating for expanding radiographic guidelines have often been written by authors with an interest in technique systems that rely on radiographic screening of patients, demonstrate methodological flaws, and have been refuted in the literature [30–37]. The current study seems to indicate that the justifications some chiropractors use are based in fear of medicolegal implications or uncertainty about skills with history taking and physical exam procedures. This is evidenced by the justifications such as ‘looking for anomalies’, ‘never been x-rayed before’, and ‘minimal trauma’.

All the technique systems listed by respondents in Items 7, 12, and 30 require radiography as part of their paradigm for subluxation analysis with one exception. Diversified is a word with various meanings in chiropractic, but is probably most commonly understood to be a set of treatment techniques taken from different sources. It does not encompass a rigid system of analysis; there is not a requirement for radiographic subluxation detection associated with it [12]. However, the Medicare system in the USA requires ‘subluxation’ to be diagnosed, and until 2000 Medicare required radiography for the diagnosis [38]. So evidence-based practitioners who use a diverse group of manual treatment methods may call their technique ‘Diversified’. However, they would not adhere to a technique-specific protocol for patient evaluation, diagnosis and treatment, and are simply forced to use the word ‘subluxation’ in their diagnosis in order to be reimbursed under these schemes.

Although only a small minority of respondents reported knowledge of routine post-treatment radiography by referring chiropractors or teaching institutions, the practice still seems to exist. This is arguably the least justified use of radiography, in that the ‘post’ radiographs have no clinical use. They are essentially just a sales tool that chiropractors use to show patients that they have replaced bones to their ‘correct’ locations [19, 39].

Full-spine radiography, sometimes called ‘spinography’ in chiropractic, is associated with several techniques systems that require x-ray screening, involving the use of ionising radiation without clinical justification [12]. Some of the inappropriate justifications for radiography cited by respondents relate to these techniques as well as chiropractic tradition, and lack of confidence with clinical diagnostic skill. Examples of responses include ‘Full spine films on every patient without regard for symptoms or age’, ‘areas imaged do not correlate to symptoms’, ‘3 region subluxations’, and scoliosis assessment either after skeletal maturity or with no clinically visible evidence of curvature of the spine. Inappropriate full-spine radiography is particularly concerning. It denotes irradiation of a large, central body area. Unless careful shielding and filtering protocols are used, sensitive areas like the gonads, breasts, and thyroid can receive large doses.

The current study revealed that non-evidence-based justifications cause chiropractic radiologists to operate within an area of ethical dilemma and this study indicates that most of them are aware of the problem. The majority of respondents indicated that they reported on images for chiropractors that they knew radiographed all or nearly all of their patients. Only nine respondents chose the option ‘I don’t have a problem with this practice’. Twenty-one tried raising the issue with the referrers, with some degree of success reported in reducing unnecessary radiography. Only two respondents reported such practices to licensing/registration/public health boards. The majority of respondents indicated some level of resignation to the situation or disclaimed responsibility because they were uncomfortable questioning another health professional’s clinical acumen. It could be argued that statistically it is highly unlikely that any referring chiropractor radiographing all patients is finding genuine justifications for the use of ionising radiation, so it would seem that issues other than professional respect are at work.

Chiropractors have a history of being attacked by the medical community. This has led to a siege mentality, a tendency to eschew anything ‘medical’ or ‘scientific’ and a lack of self-criticism [29, 40]. In addition, there is a social stigma to becoming an ‘informer’ in one’s own community. Finally, there is an inherent conflict of interest in a fee-for-service model. Fee-for-service generally means that more work generates more money. In the case of physicians, the more services they can bill for, the more money they can make. This has led to over-ordering of diagnostic tests, over-provision of treatment services, and over-prescription of pharmaceuticals [41–45]. These practices run in obvious contradiction to the ethical provision of professional healthcare services. Countries with national health services or private health provision services in which physicians are employed on fixed salaries avoid this inherent conflict. However, most chiropractors, including DACBRs, work in a fee-for-service model. The financial interest of DACBRs lies in reporting on as many imaging studies from as many referring chiropractors as possible. Therefore, it is against a DACBR’s financial interest to act ethically in the interest of the referring chiropractor’s patients. By encouraging referrers who may radiograph all their patients to radiograph fewer, they advocate reducing their own business. In addition, although radiographic subluxation analysis is not a reportable offence in and of itself, the use of ionising radiation is regulated in many parts of the world, and its use without proper justification according to accepted guidelines is an offence, reportable to regulatory bodies. This creates a similar dilemma to the previous one. If a DACBR reports a referring chiropractor for inappropriate use of ionising radiation, the DACBR risks losing the referrer entirely. These factors may lead DACBRs to ignore potentially unethical or illegal situations, or rationalise taking no action by giving referrers more benefit of the doubt than they may deserve. This dilemma is not unique to chiropractic radiologists; many healthcare professionals working in fee-for-service systems have a financial interest in ordering more diagnostic tests and giving more treatment.

About a quarter of respondents reported that they had been asked by chiropractors to utilise a radiographic subluxation analysis system. Most declined either outright or by deflecting the question, but two acceded. This ties in with a theme that emerged from the last item to the survey, that is, some DACBRs think the future of chiropractic radiology lies in reinforcing the differences between them and medical radiologists, and more broadly, between chiropractors and medical doctors. This, and other ideas raised by respondents will be explored in more detail in the second paper in this two-paper series.

### Limitations

At 38%, the response rate was low. However, a recent meta-analysis of physician survey response rates reported an average of 53% [46]. Another study specifically of internet-based surveys of physicians found that response rates below 20% were not uncommon [47]. Braithwaite used five reminders to achieve a 52% response rate [48]. Due to time limitations, this study only used two reminders, and unfortunately only nine additional questionnaires were received in hard copy at the ACCR conference, possibly because only one announcement was made publicising its availability during the conference. Given the comparative data from other surveys of similar populations, the response rate for this current survey seemed to be within an expected realm. Even though the results may not be generalizable to all chiropractic radiologists, this study revealed that the historical paradigm for radiography in chiropractic persists, and not just within a tiny minority of the profession. Indeed, some chiropractic radiologists use radiographic chiropractic subluxation systems. This information is more important than the accuracy of a prevalence calculation. However a prevalence calculation would be useful, especially if it compared data across countries.

Another limitation is that chiropractic radiologists are underutilised; particularly in the USA, many chiropractors take their own radiographs, only occasionally referring images for diagnosis. Other chiropractors refer to medical imaging centres for radiographs. As such, the results of this survey are likely to underestimate the numbers of chiropractors who use radiography for subluxation analysis.

Respondents did not complete all items, in a seemingly random fashion, and the reason why is not known. It is possible that they did not wish to provide some data because of the risk of being identified from the limited sample population. It is also possible that there were technical difficulties. Perhaps the questionnaire was too complex and respondents experienced loss of concentration while answering it. In any case, it seems as if respondents simply answered the items that they wanted to, or those that they thought applied to them. Henderson also observed this phenomenon in a recent survey of DACBRs [49]. A future study could gain more detailed information with the use of focus groups or interviews.

For the time during which the first 30 responses were submitted, there was a misprint on the internet version of the questionnaire, directing respondents to proceed ahead several items. It was noticed and corrected before the 31^st^ response was submitted. The error was on Item 10; it said ‘if no, skip to question 22.’ It should have said ‘skip to question 13.’ Thirty-three respondents skipped Item 10 itself. In addition, the response rates to the items between item 13 and item 21 varied greatly. The least-skipped item was Item 19 with 8; the most skipped was Item 14 with 63. Table 10 shows the response rates for Items 13–21.

**Table 10:** Answered and skipped items that most of the first 30 respondents should have skipped if they strictly followed directions

Item number	Number of respondents who answered	Number of respondents who skipped
13	23	41
14	1	63
15	2	62
16	23	41
17	1	63
18	2	62
19	56	8
20	22	42
21	3	61

This fits with the previous observation, which noted that it seemed as if respondents completed the items that they thought applied to them or those they felt like answering, skipping the rest and not paying much attention to the directions to ‘skip ahead’ when requested. This would seem to indicate that the typographical error did not have much effect on the overall result.

There may have been more than one DACBR at a given institution leading to an overestimation of the data for the teaching institutions. A survey designed to mitigate this confounding factor would be useful in the future in order to better determine the teaching and practicing methods at the schools. In addition, this study did not distinguish between full- and part-time in each category. Therefore some of the findings may have been underestimated in terms of their prevalence in practice.

## Conclusions

The findings demonstrate an extant minority of chiropractors failing to adhere to mainstream guidelines in the use of diagnostic ionising radiation for a variety of reasons, including a continuing belief in radiographically visible chiropractic subluxations. This situation is sustained in part due to the reticence of other chiropractors to report these practices to licensing and registration boards. Investigation into other structures supporting a vitalistic belief system over evidence in chiropractic is recommended. In addition, it may be useful to explore remunerative systems that move beyond the inherently conflicted fee-for-service model.

## Additional files

Additional file 1: Survey Questionnaire. (DOCX 64 kb)

Additional file 2: Item 28: Rationales for dealing with referring chiropractors who are known to image all or nearly all their patients – answers written in the text box. (DOCX 109 kb)
