# Supplementary material for: Historical influence on the practice of chiropractic radiology: Part I - a survey of Diplomates of the American Chiropractic College of Radiology
Source: Chiropr Man Therap. 2017 May 8;25:14. doi: 10.1186/s12998-017-0146-y (PMC5421324; doi:10.1186/s12998-017-0146-y)
Supplement: Supplementary file 1 — Survey Questionnaire. (DOCX 64 kb) [file 12998_2017_146_MOESM1_ESM.docx]

**Additional file 1**

**Survey Questionnaire**

*Historical influence on the practice of chiropractic radiology and its implications for the future: a survey of the profession.*

**Please circle your answer or provide comments as appropriate**

**Demographics**

**1. Sex**

1. Female
2. Male
3. Other

**2. Age**

1. Under 25
2. 25-34
3. 35-44
4. 45-54
5. 55-64
6. 65 and over

**3. Where do you live?**

1. Australasia
2. Europe
3. North America
4. United Kingdom

**Teaching**

**4. Are you currently employed in any capacity by a chiropractic teaching institution?**

1. Yes (go to next question)
2. No (go to question 19)

**5. Does anyone at your chiropractic teaching institution currently teach any chiropractic radiographic subluxation analysis system as part of the school’s curriculum?**

1. Yes (go to next question)
2. No / unsure (go to question 8)

**6. By whom is (are) the system(s) taught at your institution?**

1. Self
2. Other chiropractic radiologists
3. Technique instructors
4. Clinic supervisors
5. Adjunct/casual faculty/staff
6. Other (specify)

**7. Please list the system(s) taught and, if known, give a brief rationale on why the particular system(s) were selected to be included in the curriculum.**

**8. Have you ever refused a request to teach a chiropractic radiographic subluxation analysis system in a chiropractic teaching institution?**

1. Yes (go to next question)
2. No (go to question 10)

**9. Please give a brief explanation on why you refused to teach a chiropractic subluxation analysis system and state the outcome of this decision.**

**10. Does your chiropractic teaching institution currently perform any chiropractic radiographic subluxation analysis systems on patients in its clinic?**

1. Yes (go to next question)
2. No (go to question 13)

**11. By whom is the use of chiropractic radiographic subluxation analysis systems on patients in your institution’s clinic?**

1. Self
2. Other chiropractic radiologists
3. Technique instructors
4. Clinic supervisors
5. Adjunct/casual faculty/staff
6. Other (specify)

**12. Please list the system(s) used and, if known, give a brief rationale on why the particular system(s) have been selected.**

**13. Does anyone at your chiropractic teaching institution currently teach the use of post-adjustment or post course-of-chiropractic-care radiographs as part of the curriculum?**

1. Yes (go to next question)
2. No / unsure (go to question 16)

**14. By whom is the use of post-adjustment or post course-of-chiropractic-care radiography taught at your institution?**

1. Self
2. Other chiropractic radiologists
3. Technique instructors
4. Clinic supervisors
5. Adjunct/casual faculty/staff
6. Other (specify)

**15. If known, please state the clinical rationale for including in the curriculum the use of post-adjustment or post course-of-chiropractic-care radiographs.**

**16. Does anyone at your chiropractic teaching institution currently perform post-adjustment or post course-of-chiropractic-care radiographs on patients in its clinic?**

1. Yes (go to next question)
2. No (go to question 19)

**17. By whom is the use of post-adjustment or post course-of-chiropractic-care radiography used on patients at your institution’s clinic?**

1. Self
2. Other chiropractic radiologists
3. Technique instructors
4. Clinic supervisors
5. Adjunct/casual faculty/staff
6. Other (specify)

**18. If known, please state the clinical rationale for performing post-adjustment or post course-of-chiropractic-care radiographs on patients in the institution’s clinic.**

**Clinical practice**

**19. Do you currently work in clinical practice treating patients directly?**

1. Yes (go to next question)
2. No (skip to question 22)

**20. Do you currently use any chiropractic radiographic subluxation analysis systems in your clinical practice?**

1. Yes (go to next question)
2. No (go to question 22)

**21. Which system(s) do you use and what is the rationale behind using it (them)?**

**Radiology Practice**

**22. Do you currently work in radiology practice, reporting on diagnostic images for chiropractors or other health care providers?**

1. Yes (go to next question)
2. No (skip to question 34)

**23. Do you report on full spine images? These may be in any format: sectional, digitally stitched, or single film.**

1. Yes (go to next question)
2. No (skip to question 25)

**24. What percentage of your practice is comprised of reporting full spine images?**

1. 80-100%
2. 60-79%
3. 40-59%
4. 20-39%
5. <20%

**25. Regarding justification for the use of ionizing radiation, what percentage of the patients referred to you reflects the use of mainstream radiographic guidelines?**

1. 80-100%
2. 60-79%
3. 40-59%
4. 20-39%
5. <20%

**26. What are some of the justifications you see on referral forms that do NOT reflect the use of mainstream radiographic guidelines?**

**27. Do you currently, or have you ever, reported on images for chiropractors that you know take radiographs on all or nearly all of their patients?**

1. Yes (go to next question)
2. No (skip to question 29)

**28. Reporting on the images of chiropractors who radiograph all their patients may present an ethical dilemma to the reporting chiropractic radiologist. How do you deal with this issue? (Tick all that apply)**

1. I don’t have a problem with this practice.
2. I know that the images are at least being properly scrutinized for pathology.
3. It’s not my place to question another chiropractor’s clinical judgment
4. I do not speak with or examine the patients, so I’m not in a position to pass judgment.
5. It’s just part of the business I’m in.
6. I have raised the issue with the referring chiropractors, but none have changed.
7. I have raised the issue with the referring chiropractors, and have helped reduce this practice.
8. I have reported such practices to licensing/registration/public health boards.
9. Other (specify, or elaborate on any of the above-ticked items)

**29. Do you currently use any chiropractic radiographic subluxation analysis systems in your radiology practice?**

1. Yes (go to next question)
2. No (skip to question 31)

**30. Which system(s) do you use and why?**

**31. Have you ever been asked to use a chiropractic radiographic subluxation analysis system by a referrer? How did you respond?**

1. Yes - please comment below
2. No

**32. Do you report on post-adjustment or post course-of-chiropractic-care radiographs (as far as you are aware?)**

1. Yes (go to next question)
2. No (skip to question 34)

**33. What percentage of your reports are for post-treatment or post course-of-chiropractic-care radiographs?**

1. 80-100
2. 60-79
3. 40-59
4. 20-39
5. <20

**34. What do you think the future holds for the practice of chiropractic radiology? Please feel free to comment at length, or elaborate on answers to any of the above questions.**
