# Supplementary material for: Historical influence on the practice of chiropractic radiology: Part I - a survey of Diplomates of the American Chiropractic College of Radiology
Source: Chiropr Man Therap. 2017 May 8;25:14. doi: 10.1186/s12998-017-0146-y (PMC5421324; doi:10.1186/s12998-017-0146-y)
Supplement: Supplementary file 2 — Item 28: Rationales for dealing with referring chiropractors who are known to image all or nearly all their patients – answers written in the text box. (DOCX 109 kb) [file 12998_2017_146_MOESM2_ESM.docx]

**Additional file 2**

**Rationales for dealing with referring chiropractors who are known to image all or nearly all their patients – answers written in the text box**

NOTE: Responses have been edited for spelling and grammar as well as to maintain anonymity without changing meaning.

- My job is to read the images that are sent to me. The clinician decides why the images need to be taken.
- Extreme cases I have reported and refuse to read for these folks, particularly when young patients are routinely exposed. I refuse to read for people who do not take my advice, at least somewhat, regarding caution with over irradiating patients.
- I have reported one chiropractor to a state board who did pre/post adjusting x-rays multiple times on a patient who later came to see me for treatment. My complaint was not acknowledged and I don't believe any action was taken. I do think the risk to public for missed DX on x-rays not reviewed by a radiologist is greater than radiation risk to public. I also think chiropractors who put force into the spine are justified to image what they push on when MDs who write scripts are not... Current guidelines are more aligned with medical practice than chiropractic practice. [Non-essential information removed to preserve anonymity.]
- It's complex and unless there is a chiropractic board scope of practice violation then nothing will change. A complaint will just irritate the client, reduce or eliminate radiologist referrals and consults and maybe not have films read at all. In addition, even Dr [name removed to preserve anonymity] and I could disagree on when to image someone. We have limited information, don't see the patient and vary based on practice experience. As long as it is not violating practice standards that variation will remain and rightly so.
- Only a small percentage of chiropractors in this country do this. The vast majority follow evidence-based imaging guidelines.
- I have helped reduce this practice in some cases, while others have ignored my advice. I also teach students about the perils of this practice to try to prevent it from the beginning.
- I occasionally will contact and question the necessity of radiograph but generally most of the referring physicians referring to our imaging centers seem to have good clinical criteria and judgment. 80% of my reads are for DCs and the remaining 20% are MDs and DOs.
- We have some clients whom I suspect take x-rays on all of their patients, although I don't know this with certainty. We don't have any clients who send all of their films in for review.
- Some of the practices I read for are strictly personal injury.
- My main concern is x-ray quality. I am not in a position to change whether a patient is imaged or not because I am not there upon the examination. It is very important to me that image quality is optimum so a proper analysis can be made of the film study.
- I'm also working with the imaging centre (a national chain) to develop a strategy to help educate chiropractors about proper x-ray justification and guidelines.
